# 836. Coccidioidomycosis in the Central U.S. – A Retrospective Case Series

**DOI:** 10.1093/ofid/ofad500.881

**Published:** 2023-11-27

**Authors:** Brian M Scott, Olivia Gordon, Joseph Sassine, Syeda Sahra, Deidra J Bowman, Nelson I Agudelo Higuita

**Affiliations:** University of Oklahoma Health Sciences Center, Oklahoma City, Oklahoma; University of Oklahoma Health Sciences Center, Oklahoma City, Oklahoma; University of Oklahoma Health Sciences Center, Oklahoma City, Oklahoma; Oklahoma University of Health Sciences Center, Oklahoma City, Oklahoma; University of Oklahoma Health Sciences Center, Oklahoma City, Oklahoma; University of Oklahoma Health Sciences Center, Oklahoma City, Oklahoma

## Abstract

**Background:**

Known as Valley fever, coccidioidomycosis is a disease endemic to arid regions of the Western Hemisphere. In the Southwestern US, Coccidioides spp. may account for up to 20-25% of cases of community acquired pneumonia. Clinical manifestations vary widely, from asymptomatic infection to life-threatening disease, especially in immunocompromised hosts. Management can prove to be challenging, especially in disseminated disease; however, the overall mortality rate remains low. We describe the results of a single-center, retrospective case series over the last 20 years.

**Methods:**

A list of patients was identified from medical records, pathology, and microbiology, utilizing diagnosis codes and the key words ‘coccidioidomycosis’ and ‘valley fever’. Chart review was conducted on each of these cases. Variables included patient demographics, comorbidities, travel to an endemic area, site of infection, diagnosis, treatment, and outcomes.

**Results:**

A total of 33 patients were identified using the methods above; however, only 26 patients were included in our study. 7 patients were excluded due to limited medical records. Most patients identified were male, middle-aged, and Caucasian (table 1). The most common comorbidity was diabetes, and the most common site of infection was pulmonary, followed by central nervous system (table 1, table 2). Nearly all patients received systemic antifungals, and about half underwent surgery for treatment (table 2).

**Figure:**
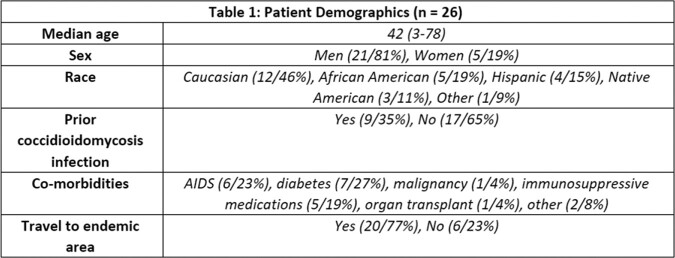

**Figure:**
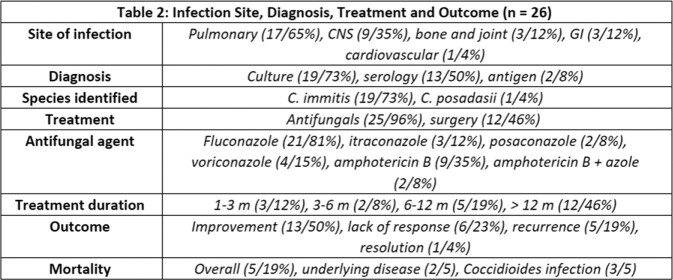

**Conclusion:**

To our knowledge, this is the largest single-center case series of coccidioidomycosis from a non-endemic area. Although most of our patients had visited an endemic area, several cases may be native to Oklahoma, considered a non-endemic region, based on available information in the medical record. Diabetes mellitus was the most frequent comorbidity exhibited by our patient population supporting surveillance data of its association with severe disease. Compared to other case series of coccidioidomycosis, our patient population had higher rates of immunocompromise and had both a higher overall mortality and rate of disseminated disease.

**Disclosures:**

**All Authors**: No reported disclosures

